# The Many Faces of Precarity: Research on Social Security and Diverse Populations

**DOI:** 10.1093/geroni/igaf122.1516

**Published:** 2025-12-31

**Authors:** Jennifer Kaufman, Siavash Radpour

## Abstract

The New York Retirement and Disability Research Center (RDRC) was one of two new RDRCs created by a cooperative agreement with the Social Security Administration in 2023. The center aimed to focus on structural health, wealth, and life course processes that yield both opportunities and social and economic stratification. Although the center was defunded in early 2025, its first year of work brought together public health professionals, demographers, sociologists, urban planners, economists, and gerontologists to study a breadth of populations using both quantitative and qualitative methods. This symposium presents a range of work that examines how people get by in the face of disability, disaster, and aging on a low income. Our first presentation employs a new poverty measure to illuminate the effects of homeownership, health and social insurance benefits, and other transfers (especially rental assistance) on poverty among older adults. Our second presentation investigates the work and income situations of older adults displaced by disasters. Our third presentation examines how Social Security disability decisions affect health insurance status, poverty, and out-of-pocket medical costs. Our final presentation is a qualitative study exploring the experience of formerly incarcerated older adults in applying for disability benefits. Findings from this symposium demonstrate the complexity of economic insecurity and highlight the role of public benefits in alleviating poverty among older adults.

